# Diagnosis of pediatric mitochondrial diseases via targeted next-generation sequencing (NGS): real-world data with the Blueprint Genetics^®^ platform

**DOI:** 10.1186/s13023-026-04213-9

**Published:** 2026-04-09

**Authors:** Annamaria Sapuppo, Grete Francesca Privitera, Vincenzo Sortino, Piero Pavone, Agata Polizzi, Martino Ruggieri, Raffaele Falsaperla

**Affiliations:** 1 Unit of Pediatrics and Pediatric Emergency, Azienda Ospedaliero-Universitaria Policlinico “Rodolico-San Marco”, San Marco Hospital, University of Catania, Catania, 95121 Italy; 2 Department of Clinical and Experimental Medicine, Bioinformatic Unit, University of Catania, 95123 Catania, Italy; 3 National Council of Research, Institute for Research and Biomedical Innovation (IRIB), Catania, Italy; 4 PhD Program in Innovative Technologies in Biomedical Sciences, University Kore of Enna, Enna, Italy; 5 Unit of Pediatric Clinic, Azienda Ospedaliero-Universitaria Policlinico “G.Rodolico-San Marco”, Department of Clinical and Experimental Medicine, University of Catania, 95123 Catania, Italy; 6 Department of Medical Science-Pediatrics, University of Ferrara, Ferrara, 44124 Italy

**Keywords:** Mitochondrial diseases, Next-generation sequencing, Pediatric neurology, Diagnostic sensitivity, Blueprint genetics, Buccal swab, Clinical phenotype, Early onset

## Abstract

**Background:**

The diagnosis of mitochondrial disorders (MDs) in pediatric patients is complex and often delayed due to heterogeneous clinical presentations and limited access to invasive confirmation tests such as muscle biopsy.

**Objective:**

To evaluate the diagnostic sensitivity and specificity of a targeted next-generation sequencing (NGS) panel (Blueprint Genetics^®^ “FLEX Comprehensive Epilepsy Panel Plus”) for detecting MDs in a cohort of pediatric patients with neurological symptoms compared with gold standard tissue biopsy and clinical follow-up. The primary endpoint was the diagnostic accuracy of targeted NGS panel testing for pediatric MDs. The secondary endpoints were the identification of clinical predictors and biochemical correlations associated with increased diagnostic yield and the assessment of age-dependent diagnostic patterns.

**Methods:**

We enrolled 36 pediatric patients between May 2022 and July 2023 on the basis of predefined clinical criteria suggestive of MDs. All patients underwent buccal swab-based targeted NGS testing. Confirmatory tissue biopsies and functional studies were performed in selected cases as the gold standard reference. True positives were defined as pathogenic/likely pathogenic variants confirmed by tissue biopsy and/or strong clinical correlation. Statistical analyses included Pearson correlation, Fisher’s exact test, and univariate logistic regression, stratifying data by age of onset and clinical features. The study followed the Strengthening the Reporting of Observational Studies in Epidemiology (STROBE) guidelines to ensure methodological rigor and transparency. This was a convenience sample without a priori power calculation.

**Results:**

Five patients (5/34 out of 36 patients who completed the study, 14.7%) harbored variants in MD-associated genes. Pathogenic or likely pathogenic variants were confirmed in three cases through tissue biopsy, acting as true positives. The sensitivity was 100% (95% CI: 29.2–100%), the specificity was 94% (95% CI: 79.6–99.3%), the accuracy was 94% (95% CI: 80.3–99.3%), and the positive predictive value was 60% (95% CI: 14.7–94.7%). The positive predictive value reflects the presence of variants of uncertain significance (VUS) requiring further validation. Statistically significant correlations were observed between the presence of MD variants and elevated blood lactate (*R* = 0.51, *p* < 0.05) and GGT levels (*R* = 0.46, *p* < 0.05) and reduced hemoglobin (*R*=-0.42, *p* < 0.05). Eight specific clinical signs, occurring at < 12 months of age, strongly predicted MD-related variants with statistical significance (*p* < 0.05).

**Conclusion:**

Targeted NGS via buccal swabs demonstrates high accuracy and specificity in diagnosing pediatric MDs when guided by a structured clinical phenotype and confirmed by tissue analysis. Early onset of specific clinical signs should prompt NGS testing and may reduce unnecessary invasive procedures. Early diagnosis enables improved patient management and family counseling.

## Introduction

Mitochondrial disorders (MDs) are a heterogeneous group of metabolic diseases caused by dysfunction of the mitochondrial respiratory chain (MRC), which is essential for cellular energy production. These diseases can arise from mutations in either mitochondrial DNA (mtDNA) or nuclear genes that encode mitochondrial proteins. Pediatric presentations of MDs often include psychomotor delay, epilepsy, deafness, optic atrophy, and multisystemic involvement, frequently affecting high-energy-demand organs such as the brain, muscle, and heart [1–3]. Despite advances in our understanding of the molecular basis of MDs, diagnosis remains a significant challenge owing to clinical heterogeneity and the invasive nature of traditional diagnostic tools such as muscle biopsy.

Next-generation sequencing (NGS) has revolutionized genetic diagnostics by enabling the simultaneous analysis of hundreds of MD-related genes [4]. The Blueprint Genetics^®^ FLEX Comprehensive Epilepsy Panel Plus includes both nuclear and mitochondrial genes associated with neurological disorders, offering a promising non-invasive diagnostic alternative to tissue-based testing [5]. However, real-world data on the performance of such panels, particularly in pediatric settings, are limited.

Several critical gaps exist in the current literature regarding the NGS-based diagnosis of pediatric MDs. First, while whole exome sequencing (WES) studies have demonstrated diagnostic utility in the management of MDs, targeted panels specific to mitochondrial pathways have received less systematic evaluation in pediatric populations [6, 7]. Second, most published studies focus on adult cohorts or mixed age groups, limiting the applicability of findings to pediatric practice, where phenotypic presentations and genetic penetrance may differ significantly [3, 8]. Third, real-world performance data comparing NGS panels to traditional tissue-based diagnoses in routine clinical practice remain scarce, particularly in pediatric neurology settings.

The recent Clinical Genome Resource Mitochondrial Disease Gene Curation Expert Panel (GCEP), comprising 40 international experts in MDs, strongly advocates for a “*genomics-first*” approach in MD diagnosis, particularly in pediatric populations where invasive procedures carry greater risks and technical challenges [9, 10]. This paradigm shift emphasizes early genetic testing before tissue biopsy, potentially reducing diagnostic delays and unnecessary procedures. However, the implementation of this approach requires robust real-world evidence demonstrating the diagnostic accuracy and clinical utility of targeted NGS panels in pediatric settings.

The estimated prevalence of primary MDs in pediatric populations ranges from 1 in 5,000 to 1 in 10,000 live births globally [3]. However, precise epidemiological data for our specific catchment area (Sicily, Italy) are not available, highlighting the need for improved diagnostic protocols and disease registries.

This study aims to fill these knowledge gaps by providing real-world evidence for the diagnostic accuracy—specifically sensitivity and specificity—of a targeted NGS panel performed via a buccal swab in a pediatric cohort with clinical features suggestive of MDs, using tissue biopsy and comprehensive clinical follow-up as the gold standard reference. We also investigated whether specific signs, symptoms, and age of onset can improve the diagnostic yield and guide more targeted use of genetic testing. Early and accurate diagnosis of MDs can significantly impact patient management, enabling appropriate therapeutic interventions, genetic counseling, and improved quality of life outcomes.

## Materials and methods

### Study design and population

A single-center observational study was conducted from May 2022 to July 2023 at the Pediatric Clinic of the University of Catania. Thirty-six pediatric patients (aged 1 month to 19 years) with neurological symptoms suggestive of MDs were initially enrolled according to predefined inclusion criteria. Written informed consent was obtained from the legal guardians of all participants. The study was conducted in accordance with the Strengthening the Reporting of Observational Studies in Epidemiology (STROBE) guidelines [11]. A completed STROBE checklist for this observational study is available as supplementary material upon request from the corresponding author. This was a convenience sample without a priori sample size calculations, reflecting the exploratory nature of this real-world study.

### Inclusion criteria

Patients were eligible if they presented with at least one of the following clinical features:

- Oculomotor abnormalities.
- Dysautonomia (excessive sweating, temperature instability, digestive disorders, nasal congestion, relevant sleep disorders).
- Altered glucose metabolism (> 3 hypoglycemic episodes < 2.5 mmol/L, diabetes with poor response to insulin).
- Magnetic resonance imaging (MRI) findings (basal ganglia/brainstem involvement, leukoencephalopathy, cerebellar atrophy).
- Valproate-induced liver failure: Aspartate aminotransferase (AST)/alanine aminotransferase (ALT) ratio > 3x the upper normal limit with valproate exposure.
- Continuous partial epilepsy or nonconvulsive status epilepticus.
- Myoclonus.
- Sensori neural hearing loss, optic atrophy, or retinitis pigmentosa.
- Non-vascular cerebral infarcts.
- Elevated lactate levels in the serum (> 3 mmol/L) or cerebrospinal fluid (CSF, > 2.2 mmol/L) were detected.

### Exclusion criteria

Patients with a previously confirmed genetic/metabolic diagnosis were excluded.

### Genetic testing

DNA was extracted from buccal epithelial cells using sterile swabs. Samples were shipped to Blueprint Genetics^®^ (Helsinki, Finland) under controlled conditions maintaining sample integrity according to the laboratory’s standard operating procedures. The samples were processed with the Blueprint Genetics^®^ FLEX Comprehensive Epilepsy Panel Plus (Blueprint Genetics^®^, Helsinki, Finland) which targets 668 nuclear and 37 mitochondrial genes. Blueprint Genetics^®^ employs Illumina sequencing technology with proprietary enrichment methods, though specific platform details are considered proprietary information. The laboratory maintains ISO 15189:2012 and CAP (College of American Pathologists) accreditation, ensuring compliance with international standards for clinical diagnostic testing [5]. Variant classification followed the American College of Medical Genetics and Genomics (ACMG) guidelines (v3.2) [12].

### Gold standard definition and confirmatory testing

In our study, **true positives** were defined as patients with pathogenic or likely pathogenic variants confirmed by one or more of the following:

- Positive tissue biopsy samples (muscle or skin fibroblasts) showing mitochondrial dysfunction.
- Strong clinical correlation with established genotype‒phenotype associations.
- Functional studies confirming pathogenicity.

**True negatives** were defined as patients with negative NGS results and either negative tissue biopsy results or the absence of clinical progression consistent with MD during follow-up. The median clinical follow-up duration was 12 months (range: 6–18 months) for all patients.

Variants of uncertain significance (VUS) were classified according to ACMG guidelines when insufficient evidence existed for pathogenic or benign classification, specifically when variants met 1–2 pathogenic criteria but lacked functional validation or population frequency data [12]. In the presence of VUS, the clinical management of the patients was as follows:

1. **Baseline**: Detailed personal and family history collection, evaluation of dysmorphic features, and biochemical (lactate, creatine kinase, liver function tests, etc.) and radiological (ultrasound, MRI) assessments.
2. **Follow-up**: scheduled reassessment every 6 months, with standardized clinical scoring using revised mitochondrial diagnostic criteria (MDCs) for MD probability [13, 14]:
  **Low clinical suspicion (score < 2/8)**: Observation with periodic follow-up
  **Moderate suspicion (score 2–4/8)**: Muscle or other tissue biopsy (as a skin biopsy for fibroblast respiratory chain analysis) within 6 months
  **High suspicion (score > 5/8)**: Immediate tissue biopsy and trio analysis; functional studies
3. **Genetic counseling**: Comprehensive counseling provided at initial diagnosis and in case of reclassification.

When pathogenic or likely pathogenic variants were detected, simultaneous genetic testing of the patient and both biological parents (trio analysis) and muscle or skin biopsy were performed to determine inheritance patterns and confirm variant pathogenicity. VUS or low heteroplasmy variants prompted further biochemical testing and possible biopsy for classification.

### Statistical analysis

All the statistical analyses were conducted via R (v.4.4.0). Sensitivity was calculated as true positives/(true positives + false negatives); specificity as true negatives/(true negatives + false positives); accuracy as (true positives + true negatives)/total patients; and positive predictive value (precision) as true positives/(true positives + false positives). Confidence intervals (95% CIs) were determined via the Wilson score method. Pearson correlation, chi-square tests, and univariate logistic regression were employed to explore associations between genetic findings and clinical or laboratory variables. Statistical significance was set at *p* < 0.05. Diagnostic performance metrics were validated via the R caret package (v.7.0–1) [15], following STROBE checklist for observational studies.

## Results

### Cohort characteristics

Of the 36 patients initially enrolled [(19 females; mean age 11.5 years; 8 patients (22%) < 1 year of age], 34 were included in the final analysis. Two patients were excluded because they underwent trio-only testing, which did not align with the primary panel-based workflow. The most frequent inclusion criteria were neuroimaging abnormalities (24%), followed by dysautonomia (20%) and oculomotor disturbances (15%).

### Genetic findings and confirmation

Five patients (5/34, 14.7%) harbored genetic variants in MD-associated genes (Table 1).

- **Confirmed Pathogenic Variants** (True Positives, *n* = 3):
  1. **Patient 1 (F**,** 1 m)**: Compound heterozygous variants in the *BCS1L* gene (BCS1 homolog, ubiquinol-cytochrome C reductase complex chaperone, GRACILE syndrome, OMIM 603358), confirmed via fibroblast biopsy and Trio exome analysis. The patient died at 3 months of age.
  2. **Patient 2 (F**,** 5y)**: homozygous likely pathogenic variant in the *POLG* gene (DNA polymerase gamma, catalytic subunit, POLG-related disorder, OMIM 174763), confirmed via muscle biopsy and Trio exome analysis. Clinical follow-up revealed progressive epileptic encephalopathy.
  3. **Patient 3 (M**,** 19y)**: compound heterozygous variants in the *IBA57* gene (Iron-Sulfur cluster assembly factor, Multiple Mitochondrial Dysfunction type 3, OMIM 615330), confirmed via muscle biopsy. Clinical follow-up revealed progressive myopathy.

- **Unconfirmed Variants (*n* = 2)**:
  1. **Patient 4 (M**,** 7y)**: low level *MT-TK* (mitochondrially encoded transfer RNA, OMIM 590060) heteroplasmic variant (6.6%). Muscle biopsy was negative, and the patient’s clinical follow-up was stable.
  2. **Patient 5 (F**,** 7 m)**: low level *MT-ND4* (mitochondrial NADH-ubiquinone oxidoreductase chain 4 protein, OMIM 516003) heteroplasmic variant (5.9%). Muscle biopsy was negative, and the patient’s clinical follow-up was stable.

Additional incidental findings included pathogenic variants in nuclear genes: *CDKL5* (*n* = 1, Cyclin-dependent kinase-like 5, CDKL5 deficiency disorder with early-onset epileptic encephalopathy), *SCN2A* (*n* = 1, Sodium channel protein type 2 subunit alpha, developmental and epileptic encephalopathy), *GRIN2D* (*n* = 1, Glutamate Ionotropic Receptor NMDA Type Subunit 2D, epilepsy and intellectual disability), and *PRODH* (*n* = 1, Proline Dehydrogenase 1, hyperprolinemia) genes, These findings provided clinically relevant alternative diagnoses for 4 patients (11.8% of the cohort), demonstrating the added diagnostic value of comprehensive epilepsy panels. All patients with incidental findings were followed up with appropriate genetic counseling and targeted clinical management based on their confirmed genetic diagnosis.

**Table 1:** Five patients carrying variants of MD-associated genes

*N*°	Sex	Age at the test	Gene	MD-related genetic finding First Variant	MD-related genetic finding Second Variant	Diagnostic confirmation	Clinical Outcome at Follow-up
1	F	1 m	*BCS1L*	c.950_953del, p.(Asp317Valfs*12), **likely pathogenic**	*c.256 C > T*,* p.(His86Tyr)* **VUS**	Fibroblast biopsy + Trio exome analysis.	*Died at 3 m*
2	F	5y	*POLG*	c.2391G > T, p.(Met797Ile), **likely pathogenic.**	c.2391G > T, p.(Met797Ile), **likely pathogenic**	Muscle biopsy + Trio exome analysis	*Progressive epileptic encephalopathy*
3	M	19y	*IBA57*	*c.301_306del*,* p.(Asn101_Val102del)* **VUS**	*c.682G > C*,* p.(Val228Leu)* **VUS**	Muscle biopsy.	*Progressive myopathy*
4	M	7y	*MT-TK*	m.8328G > A, **likely pathogenic**. Heteroplasmic (6.6%)	N/A	Muscle biopsy (negative), clinical follow-up (stable)	*No disease progression*
5	F	7 m	*MT-ND4*	*m.11729T > C*,* p.(Ser324Pro)* (**VUS**). Heteroplasmic (5.9%)	N/A	Muscle biopsy (negative), clinical follow-up (stable)	*No disease progression*

Legend: A = adenine; Asn = asparagine; Asp = aspartic acid; C = cytosine; del = deletion; F = female; fs*=frameshift variant; G = guanine; His = histidine; Ile = isoleucine; Leu = leucine; M = male; m = months; Met = methionine; N/A = not applicable; Pro = proline; Ser = serine; T = thymine; Tyr = tyrosine; Val = valine; VUS = variants of uncertain significance; y = years; VUSs are reported in *italics*

### Diagnostic performance

Using tissue biopsy and clinical follow-up as the gold standard, the targeted NGS panel showed:

- **Sensitivity**: 100% (95% CI: 29.2–100%) (3/3 confirmed MD cases detected)
- **Specificity**: 94% (95% CI: 79.6–99.3%) (29/31 non-MD cases correctly identified)
- **Accuracy**: 94% (95% CI: 80.3–99.3%) (32/34 cases correctly classified)
- **Positive predictive value (PPV) (precision)**: 60% (95% CI: 14.7–94.7%) (3/5 positive NGS results confirmed)
- **The negative predictive value (NPV)**: 100% (95% CI: 88.1–100%) (29/29 negative NGS results confirmed)

The precision of 60% highlights the clinical challenge of interpreting VUS and low-level heteroplasmy in mitochondrial genes, which requires additional validation studies.

### Biochemical correlations

**Fig. 1:**
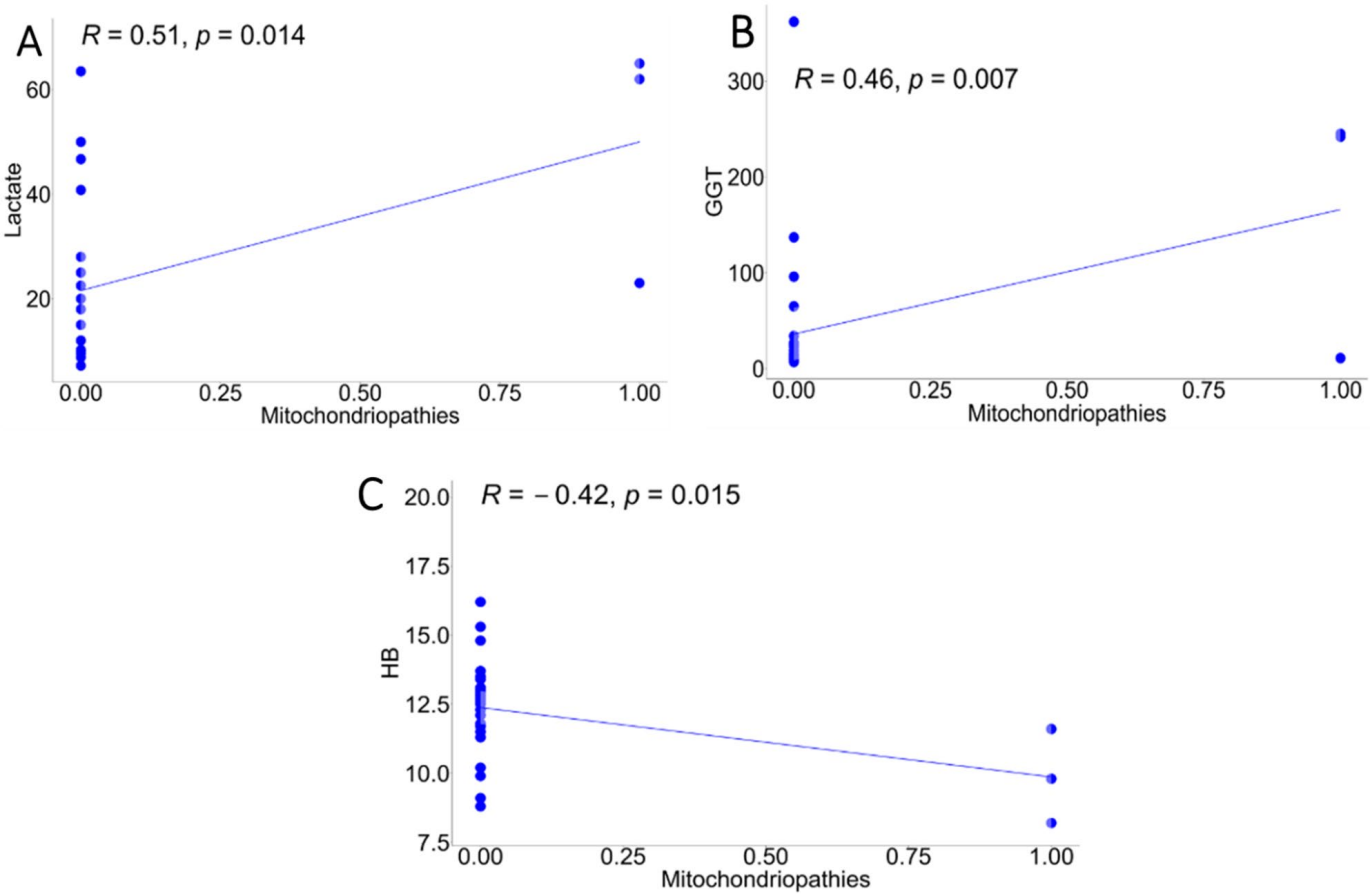
The Pearson correlation scatter plot shows the associations between laboratory variables **A**) increased lactate, **B**) increased GGT and **C**) decreased Hb in the presence of MD variants in our series

A Pearson correlation scatter plot (Fig. 1) revealed significant associations between the presence (1) or absence (0) of confirmed MD variants and the following:

- Elevated blood lactate (*R* = 0.51, *p* < 0.05)
- Elevated GGT (*R* = 0.46, *p* < 0.05)
- Decreased hemoglobin (*R* = -0.42, *p* < 0.05)

### Clinical symptoms

A correlation taking into consideration groups of variables was also performed, correlating the different signs and symptoms reported for patients carrying a genetic variant of MDs and those who did not have it. The parameters were correlated in different groups. The two most significant associations were as follows:

1. Hepatic failure due to valproate, epilepsy, visual disturbances (presents sensorineural blindness or optic atrophy or retinitis pigmentosa), increased lactate (*R* = 0.77; p value < 0.001).
2. Ataxia, visual disturbances (sensorineural blindness, optic atrophy or retinitis pigmentosa), increased lactate, episodes of respiratory difficulty/apnea, muscle weakness, and feeding difficulty (*R* = 0.78; p value < 0.001).

In both cases, we found a strong correlation.

### Clinical predictors by age of onset

**Fig. 2:**
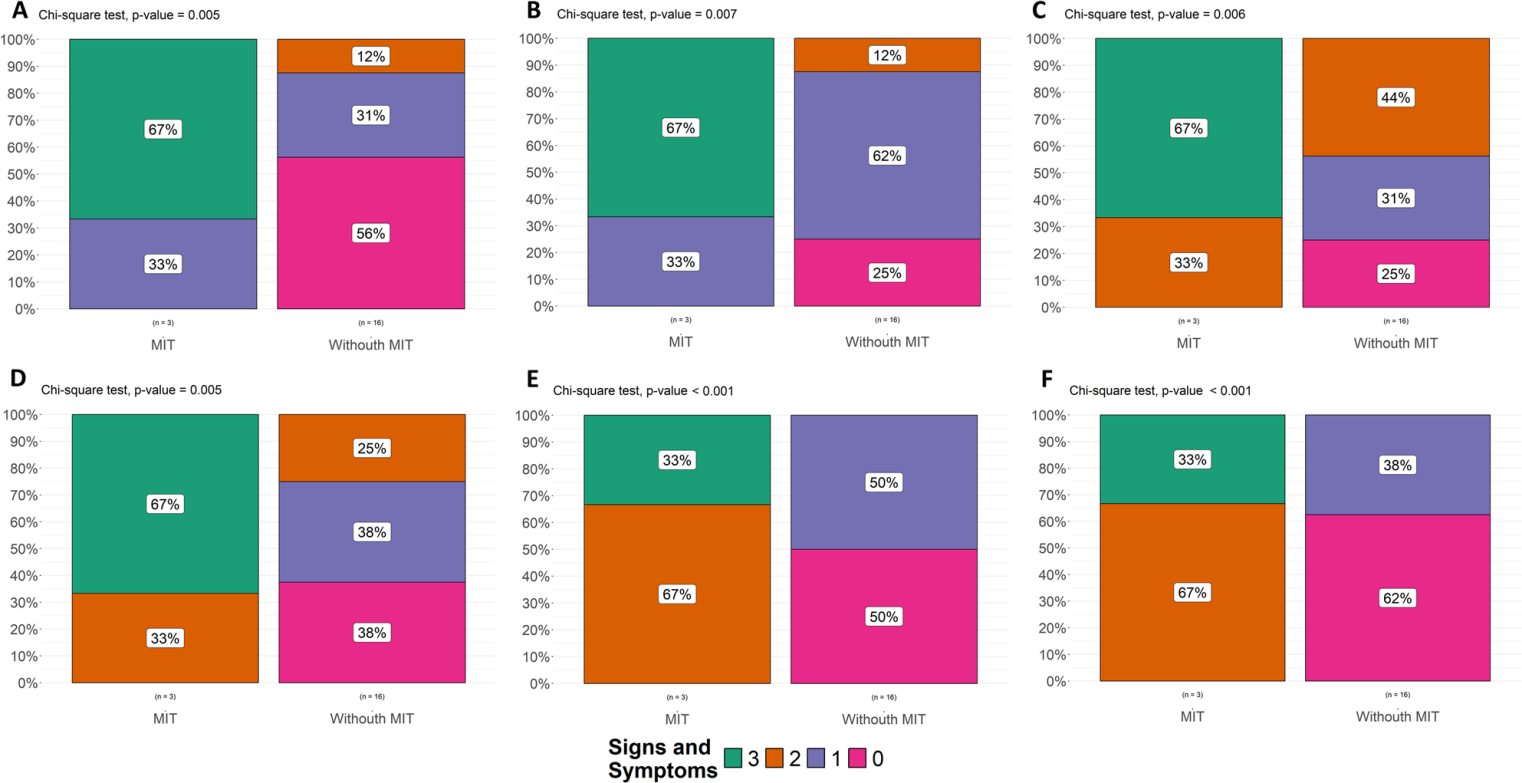
Chi square test between the presence and absence of MDs and the number of signs and symptoms. **A**) Increased blood lactate-CSF, myopathy and muscle weakness. **B**) Unjustified elevation of blood lactate-CSF, myopathy and development delay. **C**) Increased blood lactate-CSF, epilepsy and cognitive impairment. **D**) Increased blood lactate-CSF, epilepsy and muscle weakness. **E**) Unjustified increase in blood lactate-CSF, alterations in glucose metabolism and muscle weakness. **F**) Unjustified elevation of blood lactate-CSF, myopathy and alterations in glucose metabolism

Signs and symptoms were analyzed via the chi-square test, which compared the presence/absence of MD diagnosis. Patients were stratified by disease onset: 0–12 months vs. > 12 months. For the first group, 8 signs and symptoms significantly predict MD diagnosis (*p* < 0.05): (i) alteration in glucose metabolism, (ii) unjustified elevation of blood lactate/CSF, (iii) epilepsy, (iv) cognitive impairment, (v) autonomic nervous system symptoms, (vi) muscle weakness, (vii) myopathy and (viii) development delay (muscle/speech). For the group with an onset > 12 months, no individual clinical sign reached statistical significance as a predictor, suggesting the importance of early-onset phenotypes in MDs diagnosis (Fig. 2).

## Discussion

This study provides valuable real-world evidence regarding the application of a targeted NGS panel (Blueprint Genetics^®^) for diagnosing pediatric MDs. Our findings demonstrate that when combined with structured clinical phenotyping and confirmed by tissue analysis, buccal swab-based NGS achieves excellent diagnostic accuracy, with a **sensitivity of 100% (95% CI: 29.2–100%) and specificity of 94% (95% CI: 79.6–99.3%).** An overall accuracy of 94% and a PPV of 60% strongly support the use of the test as a first-tier investigation in selected pediatric populations.

Our study reinforces established concepts while identifying novel associations that warrant further investigation.

**Findings that confirm established knowledge**:

1. **High diagnostic performance of targeted NGS**: Our sensitivity of 100% (95% CI: 29.2–100%) and specificity of 94% (95% CI: 79.6–99.3%) align with published literature supporting NGS-based diagnosis for MDs, validating this non-invasive approach in pediatric populations.
2. **Lactate as a reliable biomarker**: The significant correlation between elevated blood lactate and confirmed pathogenic variants (*R* = 0.51, *p* < 0.05) reinforces its role as a key biomarker of mitochondrial dysfunction, as recommended by current diagnostic guidelines. Markedly elevated plasma lactate (> 3 mmol/L) and CSF lactate (> 2.2 mmol/L) remain critical thresholds for clinical suspicion [10, 14, 16].
3. **Early-onset phenotype significance**: Our finding that clinical signs appearing before 12 months of age strongly predict a positive genetic diagnosis confirms the well-established principle that severe, early-onset presentations often correlate with highly penetrant mitochondrial pathogenic variants.

**Novel observations requiring external validation**:

1. **GGT elevation as a potential biomarker**: The correlation between elevated GGT and MDs (*R* = 0.46, *p* < 0.05) represents a potentially novel finding. While mitochondrial hepatopathies are usually recognized as isolated liver diseases or as part of multisystem disorders [17], GGT has not been systematically evaluated as a screening biomarker for pediatric MDs. This association may reflect subclinical hepatic mitochondrial dysfunction but requires validation in larger, independent cohorts.
2. **Hemoglobin reduction**: The negative correlation between hemoglobin levels and mitochondrial genetic findings (*R*=-0.42, *p* < 0.05) suggests a possible relationship between mitochondrial dysfunction and hematological parameters. Although iron metabolism disorders are known in specific MDs [18], this association remains poorly characterized in pediatric populations and needs confirmation through prospective studies with larger sample sizes.

Historically, diagnosing MDs has been a complex and time-intensive process, primarily reliant on immunohistochemical and biochemical analyses of mitochondrial function in biopsied tissues—typically muscle or skin—considered the diagnostic gold standard [7]. Our diagnostic yield of 14.7% aligns with recent pediatric studies. Tolomeo et al. reported a 12% diagnostic yield in a 4-year pediatric cohort using targeted NGS approaches [19], whereas Heath et al. reported similar rates in their comprehensive review of pediatric mitochondrial diagnostics [20]. However, our study demonstrated superior specificity (94% vs. 85–90% in previous reports), likely reflecting our structured clinical phenotyping approach and tissue-based confirmation strategy.

While this study focused on the Blueprint Genetics^®^ platform, it is important to contextualize these findings within the broader landscape of available MDs NGS panels, such as those from Centogene^®^ (Rostock, Germany) [21], GeneDx^®^ (Stamford, USA) [22], and various academic centers with in-house mitochondrial panels. The Blueprint Genetics^®^ Comprehensive Epilepsy Panel Plus distinguishes itself through its inclusion of 705 genes (668 nuclear + 37 mitochondrial genes), which is more comprehensive than many mitochondrial-specific panels that typically include 200–400 genes. Comparative performance data across different platforms remain limited in the literature, though most validated genetic panels report similar diagnostic yields (10–20%) when applied to appropriately phenotyped patient cohorts. The choice of platform should be guided by factors including gene coverage relevant to the clinical phenotype, turnaround time, accreditation status, and institutional accessibility. Future comparative effectiveness research evaluating different NGS platforms in standardized cohorts would be valuable for optimizing diagnostic pathways and healthcare resource allocation.

Compared with historical diagnostic approaches-where step-by-step Sanger sequencing following tissue biopsy yields positive results in fewer than 10% of cases and requires months to years for completion [6]- our NGS-first approach represents a significant advancement [19]. Recent studies support shifting toward a “genomics-first” approach where NGS precedes invasive procedures [13, 20, 23]. Our study validates this approach, demonstrating that tissue biopsies can be reserved for variant confirmation and functional validation rather than serving as the initial screening tool.

The implementation of a genomics-first diagnostic algorithm could fundamentally change routine clinical practice by: (1) reducing diagnostic timelines from months or years to just weeks (2), decreasing healthcare costs by avoiding multiple invasive procedures and lengthy “diagnostic odyssey” (3), minimizing patient morbidity by reducing unnecessary biopsies in vulnerable pediatric populations, and (4) enabling earlier therapeutic interventions and family counseling.

However, the identification of specific biochemical correlations also provides potential pre-test probability tools. This is particularly relevant in low-resource settings where access to advanced sequencing or biopsy may be limited. Moreover, the finding that certain clinical combinations (e.g., visual impairment, epilepsy, and hepatic failure) are strongly associated with positive molecular diagnoses reinforces the utility of syndromic clustering in the diagnostic decision-making process. The involvement of high-energy-demand organs (central nervous system, muscle, and liver) is consistent with the pathophysiology of MDs and reflects respiratory chain compromise [24].

Our results also highlight the temporal dimension of MDs diagnosis: the earlier the symptom onset (particularly before 12 months), the greater the likelihood of ascribing a mitochondrial etiology to the clinical presentation. This finding aligns with previous literature suggesting that severe, early-onset phenotypes often correspond with nuclear or mtDNA mutations of high penetrance and pathogenicity [20].

### Clinical implications and the proposed algorithm

**Fig. 3:**
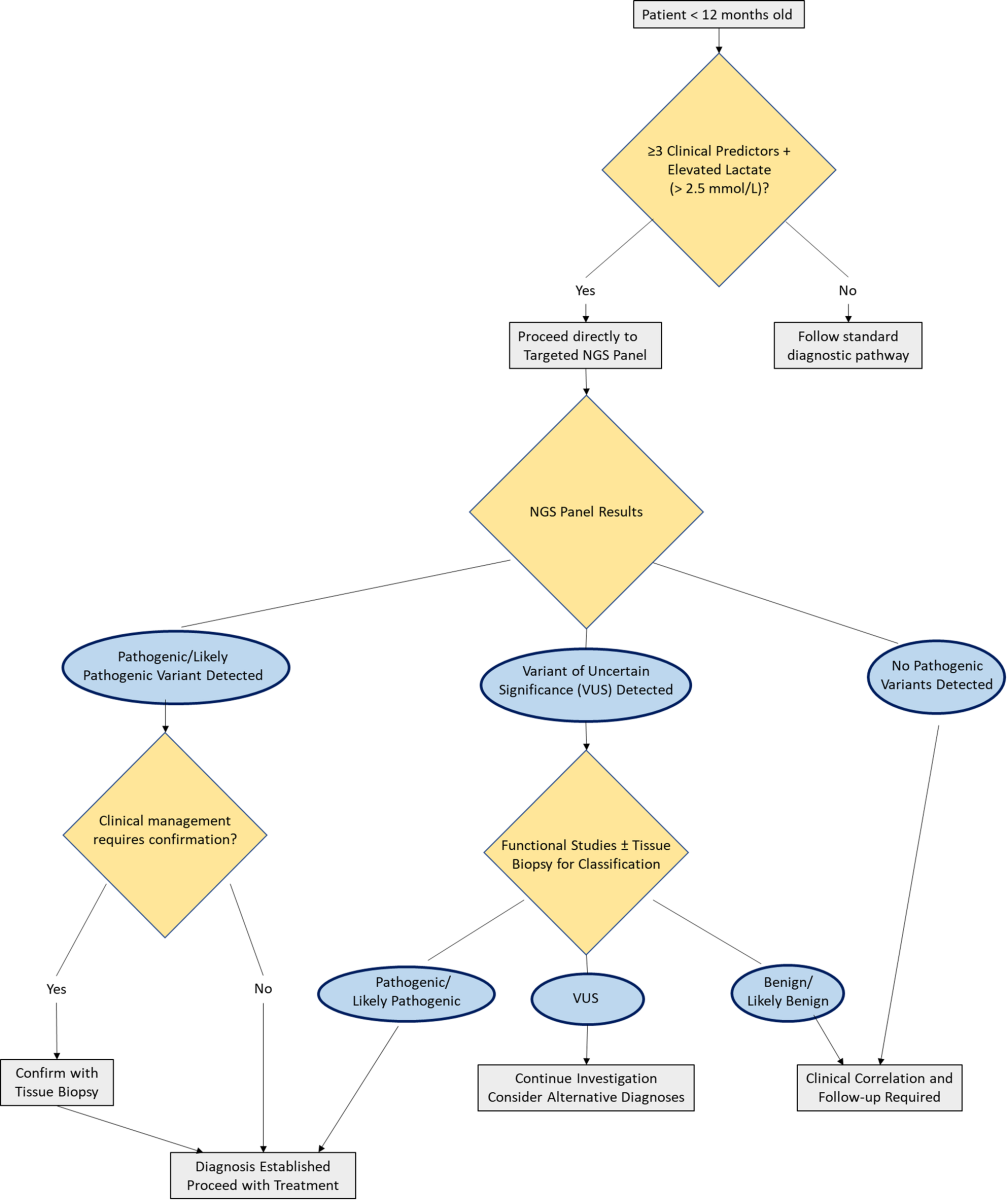
Proposed diagnostic algorithm of MDs for patients < 12 months of age

On the basis of our data, we propose an integrated diagnostic algorithm for MDs diagnosis (Fig. 3):

**- For patients < 12 months of age with ≥ 3 of the identified clinical predictors among the following**:

*(i) Alteration in glucose metabolism*,* (ii) unjustified elevation of blood lactate/CSF*,* (iii) epilepsy*,* (iv) cognitive impairment*,* (v) autonomic nervous system symptoms*,* (vi) muscle weakness*,* (vii) myopathy and (viii) developmental delay (muscle/speech).*

**+ elevated lactate (> 2.5 mmol/L):** → Proceed directly to targeted NGS panel.

→ If pathogenic/likely pathogenic variant detected → Confirm with tissue biopsy if needed for clinical management.

→ If VUS detected → Functional studies ± tissue biopsy for classification.

Our research supports this “*genomics-first*” approach, which could significantly reduce diagnostic delays and costs while minimizing unnecessary invasive procedures in vulnerable pediatric populations. Nevertheless, further multicenter studies with larger sample sizes are essential to confirm these observations and develop robust predictive algorithms that could guide testing protocols and reduce diagnostic delays.

### Study limitations

Several limitations must be acknowledged:

1. **Sample size limitations**: The small cohort (*n* = 34) limits the statistical power and generalizability of our findings. These correlations should be considered hypothesis-generating rather than definitive predictors.
2. **Single-center design**: Our results require validation in multicenter cohorts involving diverse populations.
3. **Referral bias**: Our cohort consists of patients already suspected of having MDs, potentially inflating diagnostic yield compared to unselected populations.
4. **Selection bias**: Inclusion criteria may have preferentially selected patients with more severe or classical presentations, potentially overestimating diagnostic performance.
5. **Follow-up duration**: Although the median follow-up was 12 months (range: 6–18 months), a longer period of clinical observation would further strengthen the classification of true negative cases.
6. **Tissue heteroplasmy**: Buccal swab testing may miss tissue-specific mtDNA mutations, although this limitation affects all non-invasive approaches.
7. **Statistical considerations**: Due to the exploratory nature of this study and the use of multiple comparisons, there is a potential risk of Type I error. Future studies should incorporate predictive multivariable models to better account for potential confounders and interactions among variables. External validation of these models is essential to ensure generalizability across different patient populations and clinical settings.

## Conclusion

Targeted NGS via buccal swabs should be considered a first-line diagnostic tool in pediatric patients < 12 months with suggestive phenotypes, reserving tissue biopsy for confirmation. The integration of NGS with clinical risk stratification - based on age of onset and specific biochemical markers- may optimize diagnostic efficiency while maintaining excellent accuracy. The proposed **biochemical triad** (lactate-GGT-hemoglobin) warrants validation as a pretest probability tool. This “genomics-first” approach has the potential to transform pediatric MDs diagnosis by reducing procedural burden, diagnostic delays, and healthcare costs while improving patient outcomes through earlier therapeutic intervention and family counseling. Prospective multicenter studies with larger sample sizes are essential to confirm these observations and to develop robust predictive algorithms for clinical implementation and to reduce diagnostic delays.

## Data Availability

The datasets used and analysed during the current study are available from the corresponding author on reasonable request.

## Abbreviations

A: Adenine
ACMG: American College of Medical Genetics and Genomics
ALT: Alanine aminotransferase
Asn: Asparagine
Asp: Aspartic acid
AST: Aspartate aminotransferase
C: Cytosine
CAP: College of American Pathologists
CSF: Cerebrospinal fluid
del: Deletion
F: Female
fs*: Frameshift variant
G: Guanine
GGT: Gamma-glutamyltransferase
HB: Hemoglobin
His: Histidine
Ile: Isoleucine
Leu: Leucine
M: Male
MDs: Mitochondrial disorders
Met: Methionine
MDC: Mitochondrial diagnostic criterion
MRC: Mitochondrial respiratory chain
MRI: Magnetic Resonance Imaging
mtDNA: mitochondrial DNA
m: Months
N/A: Not applicable
NPV: Negative Predictive Value
NGS: Next-generation sequencing
PPV: Positive predictive value
Pro: Proline
Ser: Serine
STROBE: Strengthening the Reporting of Observational Studies in Epidemiology
T: Thymine
Tyr: Tyrosine
Val: Valine
VUS: Variants of uncertain significance
WES: Whole exome sequencing
y: Years

## References

[1] Friedrich VK, Rubel MA, Schurr TG. Mitochondrial genetic variation in human bioenergetics, adaptation, and adult disease. Am J Hum Biol Off J Hum Biol Counc. 2022;34(2):e23629. 10.1002/ajhb.23629 34146380

[2] Lim A, Thomas RH. The mitochondrial epilepsies. Eur J Paediatr Neurol EJPN Off J Eur Paediatr Neurol Soc. 2020;24:47–52. 10.1016/j.ejpn.2019.12.021 31973983

[3] Rahman S. Mitochondrial disease in children. J Intern Med. 2020;287(6):609–33. 32176382 10.1111/joim.13054

[4] Alston CL, Rocha MC, Lax NZ, Turnbull DM, Taylor RW. The genetics and pathology of mitochondrial disease. J Pathol. 2017;241(2):236–50. 27659608 10.1002/path.4809 PMC5215404

[5] Genetic testing for epilepsy. Bluepr Genetics. https://blueprintgenetics.com/tests/panels/neurology/comprehensive-epilepsy-panel/.

[6] Neveling K, Feenstra I, Gilissen C, Hoefsloot LH, Kamsteeg EJ, Mensenkamp AR, et al. A post-hoc comparison of the utility of Sanger sequencing and exome sequencing for the diagnosis of heterogeneous diseases. Hum Mutat. 2013;34(12):1721–6. 24123792 10.1002/humu.22450

[7] Wortmann SB, Mayr JA, Nuoffer JM, Prokisch H, Sperl W. A guideline for the diagnosis of pediatric mitochondrial disease: the value of muscle and skin biopsies in the genetics era. Neuropediatrics. 2017;48:309–14. 10.1055/s-0037-1603776 28599323

[8] Rahman S, Copeland WC. POLG-related disorders and their neurological manifestations. Nat Rev Neurol. 2019;15(1):40–52. 30451971 10.1038/s41582-018-0101-0 PMC8796686

[9] Parikh S, Goldstein A, Karaa A, Koenig MK, Anselm I, Brunel-Guitton C, et al. Patient care standards for primary mitochondrial disease: a consensus statement from the mitochondrial medicine society. Genet Med Off J Am Coll Med Genet. 2017;19(12). 10.1038/gim.2017.107 PMC7804217 28749475

[10] McCormick EM, Keller K, Taylor JP, Coffey AJ, Shen L, Krotoski D, et al. Expert panel curation of 113 primary mitochondrial disease genes for the Leigh syndrome spectrum. Ann Neurol. 2023;94(4):696–712. 37255483 10.1002/ana.26716 PMC10763625

[11] Cuschieri S. The STROBE guidelines. Saudi J Anaesth. 2019;13(Suppl 1):S31–4. 30930717 10.4103/sja.SJA_543_18 PMC6398292

[12] Miller DT, Lee K, Abul-Husn NS, Amendola LM, Brothers K, Chung WK, et al. ACMG SF v3.2 list for reporting of secondary findings in clinical exome and genome sequencing: A policy statement of the American college of medical genetics and genomics (ACMG). Genet Med Off J Am Coll Med Genet. 2023;25(8):100866. 10.1016/j.gim.2023.100866 PMC10524344 37347242

[13] Witters P, Saada A, Honzik T, Tesarova M, Kleinle S, Horvath R, et al. Revisiting mitochondrial diagnostic criteria in the new era of genomics. Genet Med. 2018;20(4):444–51. 29261183 10.1038/gim.2017.125

[14] Morava E, van den Heuvel L, Hol F, de Vries MC, Hogeveen M, Rodenburg RJ, et al. Mitochondrial disease criteria. Neurology. 2006;67(10):1823–6. 17130416 10.1212/01.wnl.0000244435.27645.54

[15] Kuhn M. Building predictive models in R using the caret package. J Stat Softw. 2008;28:1–26.27774042

[16] Parikh S, Goldstein A, Koenig MK, Scaglia F, Enns GM, Saneto R, et al. Diagnosis and management of mitochondrial disease: a consensus statement from the mitochondrial medicine society. Genet Med. 2015;17(9):689–701. 10.1038/gim.2014.177 PMC5000852 25503498

[17] Alharbi H, Priestley JRC, Wilkins BJ, Ganetzky RD. Mitochondrial hepatopathies. Clin Liver Dis. 2021;18(5):243–50. 10.1002/cld.1133 PMC8605697 34840726

[18] Gao J, Zhou Q, Wu D, Chen L. Mitochondrial iron metabolism and its role in diseases. Clin Chim Acta. 2021;513:6–12. 33309797 10.1016/j.cca.2020.12.005

[19] Tolomeo D, Orsucci D, Nesti C, Baldacci J, Battini R, Bruno C, et al. The diagnostic approach to mitochondrial disorders in children in the era of Next-Generation sequencing: A 4-Year cohort study. J Clin Med. 2021;10(15):3222. 34362006 10.3390/jcm10153222 PMC8348083

[20] Heath O, Feichtinger RG, Achleitner MT, Hofbauer P, Mayr D, Merkevicius K, et al. Mitochondrial disorder diagnosis and management– what the pediatric neurologist wants to know. Eur J Paediatr Neurol. 2025;54:75–88. 39793294 10.1016/j.ejpn.2024.10.009

[21] CentoPortal^®^. https://www.centoportal.com/order/new/products/analysis-method?queryType=TEST%26query=CentoMito%2520Comprehensive.

[22] GeneDx^®^. https://providers2.genedx.com/Resources/TIS-Files/TIS-615-7.2025.pdf

[23] Chin HL, Lai PS, Tay SKH. A clinical approach to diagnosis and management of mitochondrial myopathies. Neurotherapeutics. 2024;21(1):e00304. 38241155 10.1016/j.neurot.2023.11.001 PMC10903095

[24] Gayathri N, Deepha S, Sharma S. Diagnosis of primary mitochondrial disorders -Emphasis on myopathological aspects. Mitochondrion. 2021;61:69–84. 34592422 10.1016/j.mito.2021.09.007

